# Metaplastic ossification in the cartilage of the bronchus of a patient with chronic multi-drug resistant tuberculosis: a case report

**DOI:** 10.1186/1752-1947-4-156

**Published:** 2010-05-26

**Authors:** Seok-Yong Eum, Ji-Hye Kong, Bo-Young Jeon, Sang-Nae Cho, Jhingook Kim, Laura E Via, Clifton E Barry III, Won-Jung Koh

**Affiliations:** 1 Division of Immunopathology and Cellular Immunology, International Tuberculosis Research Center, Masan, Republic of Korea; 2 Department of Microbiology, Yonsei University College of Medicine, Seoul, Republic of Korea; 3 Department of Thoracic Surgery and Department of Medicine, Samsung Medical Center, Sungkyunkwan University School of Medicine, Seoul, Republic of Korea; 4 Tuberculosis Research Section, Laboratory of Clinical Infectious Disease, National Institute of Allergy and Infectious Diseases, National Institutes of Health, Bethesda, Maryland, USA

## Abstract

**Introduction:**

Pulmonary ossification has been rarely observed in pulmonary fibrosis and in some chronic respiratory diseases such as chronic obstructive pulmonary disease. We report here a metaplastic ossification in the bronchial cartilage of a patient with multi-drug resistant tuberculosis.

**Case presentation:**

We report the case of a 41-year-old Asian man from Korea with chronic multi-drug resistant tuberculosis with a rare focus of bone formation from the cartilage of a bronchus subtending an active cavity. The patient had a large multi-lobed, thick-walled cavitary tuberculosis lesion in his left upper lobe. Severe infiltration of his lymphocytes and epithelioid cells, along with some giant cells and neutrophils, was observed in the patient's bronchial wall. Desquamated bronchial epithelium and acid-fast bacilli were found inside his bronchus. A small focus of bony metaplasia was found in the cartilage of his bronchial wall. Histopathological examination confirmed calcification and showed hematopoietic cells forming in his marrow cavity.

**Conclusions:**

Chronic inflammation in the lungs of our patient, caused by underlying tuberculosis, probably played a role in the development of osseous metaplasia from the associated cartilage of the bronchial wall.

## Introduction

Pulmonary ossification is rare and usually identified and diagnosed post-mortem by the pathologist. This phenomenon has been observed in pulmonary fibrosis and in some chronic respiratory diseases such as chronic obstructive pulmonary disease (COPD) [1-5]. We report here an unusual case of bone formation in the bronchial wall upon examination of surgically resected lung tissue from a patient with multi-drug resistant tuberculosis (MDR-TB) with chronic inflammation of the bronchial wall.

## Case presentation

A 41-year-old Asian man from Korea was referred for treatment of MDR-TB. He had been previously treated for MDR-TB with second-line anti-TB drugs for six years at another institution. Despite this treatment, his sputum smear and culture examinations were persistently positive. He used to smoke. He had a white blood cell count of 6260/μL, an erythrocyte sedimentation rate of 60 mm/h, and his C-reactive protein levels were elevated at 0.65 mg/dL. His human immunodeficiency virus antibody test was negative. On his chest X-ray examination, two cavities were observed in his left upper lobe. Acid-fast bacilli (AFB) stain revealed florid bacilli in his sputum (4+).

Meanwhile, isolation and drug susceptibility testing revealed a strain of *Mycobacterium tuberculosis *that was resistant to all first-line anti-TB drugs including isoniazid, rifampin, ethambutol, pyrazinamide, and streptomycin. Among second-line agents this strain was also found to be resistant to prothionamide, para-aminosalicylic acid, and ofloxacin. The isolate retained sensitivity to kanamycin, capreomycin, cycloserine, and moxifloxacin.

Accordingly, our patient was treated with a combination of anti-TB drugs including kanamycin, moxifloxacin, cycloserine, amoxicillin-clavulanate, and clarithromycin. Despite six months of chemotherapy, however, he still failed to convert to sputum negative status, so he was advised to undergo a left upper lobectomy and superior segmentectomy of the left lower lobe. Tissue collection from adult patients undergoing lung resection for the management of MDR-TB was approved by the Samsung Medical Center Institutional Review Board (IRB).

**Figure 1:**
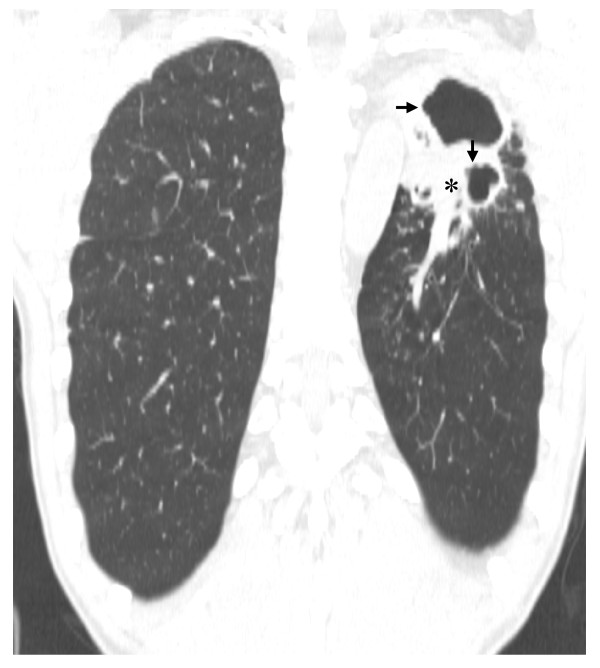
**A computed tomography scan of our patient that returned sputum-smear positive for acid fast bacilli**. He failed to achieve sputum conversion even after receiving second-line anti-TB drugs for six years. Two thick-walled independent cavities (arrows) are shown in the right upper lobe and a surrounding consolidated region is noted (asterisks).

**Figure 2:**
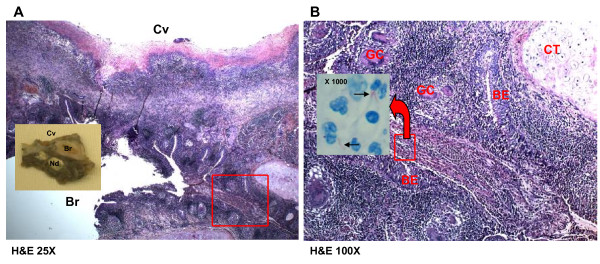
**Macroscopic and microscopic photographs showing a cavity (Cv) and an inflamed bronchus (Br)**. **(A) **Macroscopic examination of the specimen (left rectangle) shows thickening of some parts of the cavity wall and the bronchial wall. Two individual caseated nodules (Nd) are located close to the bronchus. Hematoxylin and eosin staining (original magnification, ×25) shows extensive infiltration of inflammatory cells within the cavity wall. The bronchial epithelium is severely desquamated with some intact remaining regions (right rectangle). **(B) **An enlarged photograph of right rectangle in A showing bronchial epithelium (BE) and bronchial cartilage (CT). Several multi-nucleated giant cells (GC) and severe mononuclear cells infiltration are found in the bronchial wall and Ziehl-Neelsen staining (photograph in the rectangle, ×1000) detected some *Mycobacterium tuberculosis *bacilli in caseum. (Hematoxylin and eosin staining, ×100.)

**Figure 3:**
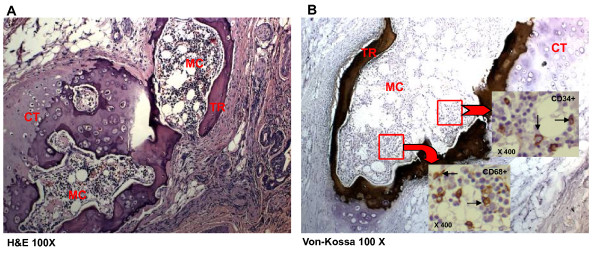
**Metaplastic bone formation in the cartilage plate (CT)**. **(A)** The newly-formed bone marrow cavity (MC) is encircled by calcified bony trabeculae (TR). This portion is further shown in B (hematoxylin and eosin staining, ×100). **(B) **Photograph shows calcified bony trabeculae (TR) stained by Von-Kossa (100). Immunohistochemistry staining detected hematopoietic CD34+ cells (arrows in upper rectangle) and CD68+ macrophages (arrows in lower rectangle) in the marrow cavity (MC).

A thick-walled lesion containing multiple cavities was observed in the left upper lobe of our patient by computed tomographic (CT) examination (Figure 1). A dilated bronchus with thickened wall and two individual granulomas with central caseation were located close to his cavity wall (Figure 2A). His bronchial epithelium was degraded and replaced with a severe inflammatory cell infiltrate. AFB were detected in the caseum inside the bronchus (Figure 2B), indicating that this bronchus was connected to the neighboring cavity. Several multi-nucleated giant cells were found in his peribronchial region (Figure 2B). Metaplastic ossification with fatty marrow replaced a portion of the cartilage plate (Figure 3A). Distribution of calcium deposits was determined by von Kossa staining and a dense band of calcium rimmed the outer edge of his marrow cavity in the cartilage plate (Figure 3B).

To further evaluate the cellular components in his marrow cavity, immunohistochemical stains were performed using antibodies to CD34 (DakoCytomation; monoclonal; N1632) and CD68 (DakoCytomation; monoclonal; 1:50). Some hematopoietic cells (CD34^+^) and macrophages (CD68^+^) were found in the marrow cavity (Figure 3B).

## Discussion

Pulmonary ossification could be defined as the histological presence of mature bone in the pulmonary parenchyma. Metaplastic pulmonary ossification is uncommon and is related to chronic lung disease. Patients are generally asymptomatic, but the condition can be associated with other pulmonary diseases such as interstitial pneumonia, fibrosis, or bronchiectasis [6]. Pulmonary ossification is not usually visible in chest X-ray. Consequently, the disease is usually discovered by chance during autopsy. High resolution computed tomography (HRCT) might be of some help in finding calcification but histopathological confirmation is required [2]. Treatment options are not well established, largely due to a lack of clinical diagnosis.

The metaplastic bones may be disseminated throughout the lungs or localized within the bronchial walls. In this case, we found a small focus of bony metaplasia in a cartilagenous plate on the bronchial wall. The bronchial cartilage is primarily responsible for maintaining the stability of the airways. Metaplastic ossification happens as part of the aging process of the thyroid cartilage [7]. Poor perfusion of the cartilage, resulting in reduced blood supply to the airways and reduced ability to control airway infection is reported to be one cause for bronchial cartilage alterations in patients who underwent lung transplantation [8]. Other reports suggest that bacterial infection associated with cystic fibrosis induces metaplastic bone replacement, as well as the destruction and elimination of the bronchial cartilage [9]. Degenerative changes in the cartilage and increased perichondrial fibrosis have been demonstrated in patients with COPD and bronchial asthma [10].

Meanwhile, there was a previous report of a patient with TB who developed metaplastic bone formation in the lung [3]. This patient also had concomitant COPD and severe fibrosis and bronchiectasis. A second case of diffuse pulmonary ossification in the alveolar septa associated with caseating granulomas was reported in a patient that had developed TB and was [4]. In the case we describe here, the observed bony metaplasia was a focal spot in the cartilage limited to a severely inflamed bronchus. This bronchus was directly adjacent to a large cavity that apparently drained caseum into the bronchial lumen and wall. The stimulation caused by this chronic inflammation might have led to the metaplastic transformation of the cartilage.

## Conclusion

Multiple factors are probably involved in the development of bony metaplasia. Given that cartilage ossifies as a result of an intense inflammatory reaction in the bronchial submucosa [9], the observations in case we describe here suggest that persistent stimulation by chronic inflammation in MDR-TB may occasionally induce the development of osseous metaplasia from the cartilage of the bronchial wall.

## Abbreviations

AFB: acid-fast bacilli; COPD: chronic obstructive pulmonary disease; MDR: multi-drug resistant; TB: tuberculosis.

## Consent

Written informed consent was obtained from our patient for publication of this case report and any accompanying images. A copy of the written consent is available for review by the Editor-in-Chief of this journal.

## Competing interests

The authors declare that they have no competing interests.

## Authors' contributions

SYE contributed to the histopathological evaluation, acquisition of data, and in conceptualizing, drafting and writing the manuscript. JHK performed histopathological and immunohistochemical examination of the specimens. BYJ assisted in the manipulation of the specimen and critically revised the manuscript. JK contributed to the operation of our patient and to the critical review of the manuscript. SNC, LV and CB contributed to research, data acquisition, and in the conception and critical revision of the manuscript. WJK contributed to the pre-operative and post-operative management of our patient and in drafting and writing the manuscript. All authors read and approved the final manuscript.
